# Changing Epidemiology of Serious Bacterial Infections in Febrile Infants without Localizing Signs

**DOI:** 10.1371/journal.pone.0012448

**Published:** 2010-08-27

**Authors:** Kevin Watt, Erica Waddle, Ravi Jhaveri

**Affiliations:** 1 Department of Pediatrics, Duke University Medical Center, Durham, North Carolina, United States of America; 2 Division of Infectious Diseases, Duke University Medical Center, Durham, North Carolina, United States of America; Columbia University, United States of America

## Abstract

**Objective:**

Historically, management of infants with fever without localizing signs (FWLS) has generated much controversy, with attempts to risk stratify based on several criteria. Advances in medical practice may have altered the epidemiology of serious bacterial infections (SBIs) in this population. We conducted this study to test the hypothesis that the rate of SBIs in this patient population has changed over time.

**Patients and Methods:**

We performed a retrospective review of all infants meeting FWLS criteria at our institution from 1997–2006. We examined all clinical and outcome data and performed statistical analysis of SBI rates and ampicillin resistance rates.

**Results:**

668 infants met criteria for FWLS. The overall rate of SBIs was 10.8%, with a significant increase from 2002–2006 (52/361, 14.4%) compared to 1997–2001 (20/307, 6.5%) (p = 0.001). This increase was driven by an increase in *E. coli* urinary tract infections (UTI), particularly in older infants (31–90 days).

**Conclusions:**

We observed a significant increase in *E. coli* UTI among FWLS infants with high rates of ampicillin resistance. The reasons are likely to be multifactorial, but the results themselves emphasize the need to examine urine in all febrile infants <90days and consider local resistance patterns when choosing empiric antibiotics.

## Introduction

The management of infants <90 days with fever without localizing source (FWLS) has been a source of much controversy and debate for the last 30 years. While the majority of these infants will only have a minor viral or bacterial infection, the literature reports that approximately 12% in those aged <30days and 9% in those 30–90 days will have a serious bacterial infection (SBI), such as bacteremia, meningitis, or urinary tract infection (UTI) [1], [2], [3], [4]. In order to better predict those infants at risk for SBI, Dagan *et al* evaluated a combination of clinical and laboratory data (no focal exam, white blood cell count (WBC) between 5,000 and 15,000/mm^3^, band forms <1500/mm^3^, normal urinalysis (UA)) to identify low and high risk groups in what would become the Rochester Criteria [5]. Modified criteria have followed that were effective at identifying low risk infants, but differed in the exact data included which resulted in inconsistent implementation [6], [7], [8], [9], [10], [11].

Several changes in the last 20 years have significantly altered the epidemiology of SBI in neonates. Group B *Streptococci* (GBS) and *E coli* have traditionally been the most important pathogens in this age group. Institution of culture-based screening and prophylaxis for GBS [12], [13], [14] has significantly lowered the incidence of this pathogen. Additionally, several authors have noted possible increased ampicillin resistance rates among pathogens causing SBI in this age group [15], [16]. In light of these changes, we conducted this study of all infants less than 3 months of age with FWLS over the last 10 years. The study questions were: What is the current frequency and distribution of SBIs in these infants and has this changed over time? What are the current rates of antibiotic resistance in pathogens identified in these patients? How do practitioners manage these patients?

## Methods

The study was conducted at Duke University Hospital, a large, tertiary care hospital in Durham, NC. Physicians in the Duke Emergency Department (ED) see over 5500 children per year who are less than 3 years of age. This project was reviewed and approved by the Duke University Institutional Review Board. A waiver of informed consent was obtained for this study because this was a retrospective study examining hospital records containing data derived for the purposes of clinical care.

### Patient Identification

Using the Clinical Microbiology laboratory database, we identified all children less than 90 days of age seen in the emergent setting that had a blood culture performed from 1997–2006. After careful chart review, we identified those infants meeting criteria for FWLS and performed further analysis. Patients were considered febrile if they had a history or examination temperature of 38.0°C or higher. Temperatures were measured rectally in the ED, and we included a fever ≥38.0°C taken by the parents that was considered reliable by the ED provider. Exclusion criteria were significant underlying illness or past medical history (PMH), subjective reports of “feeling warm” without a temperature taken, ill appearance, localizing source of infection after a thorough physical examination, or incomplete medical records. PMH could include history of immunodeficiency, previous hospitalization, significant congenital anomaly, or current antibiotic use. Complete records included documented fever, presenting symptoms, physical findings, and culture results.

Records were reviewed using the electronic medical record including ED notes, discharge summaries, information on prior and subsequent visits, and all laboratory and radiologic tests. For each patient we recorded demographic information, pertinent history, physical exam, and test results.

### Definition of SBIs

Blood culture isolates were considered pathogens if the organism is known to cause disease in healthy infants: *Escherichia coli*, *Staphylococcus aureus*, *Streptococcus pneumoniae*, *Enterococcus* sp., *Streptococcus agalactiae* (GBS), *Enterobacter cloacae*, *Klebsiella* sp., *Pasteurella* sp., *Moraxella* sp., and *Citrobacter* sp. Organisms that were considered contaminants included coagulase-negative *Staphylococci*, *Bacillus* sp., *Diptheroids*, and *Streptococcus viridans*. Organisms isolated from cerebrospinal fluid (CSF) were considered pathogens if the organism was known to cause meningitis. To separate colonization from infection, urine cultures were considered positive if a known pathogen was isolated from a catheterized specimen with >100,000 colony forming units (CFUs)/mL or 10,000–100,000 CFUs/mL with an abnormal urinalysis (UA) or in a clean catch specimen if there were >100,000 CFUs/mL and an abnormal UA. An abnormal UA was defined as positive leukocyte esterase, nitrite, or ≥10 white blood cells (WBC)/hpf and with less than 10 squamous epithelial cells/hpf. We analyzed all clinical outcomes, initial choice of antibiotics, as well as any subsequent changes made as a result of bacterial resistance or change in patient's clinical status.

### Assignment of Low Risk/High Risk Status

Infants were classified as either low risk (LR) or high risk (HR) based on historical, clinical and laboratory data. We applied the practice guidelines of Baraff to our patients [11], [17]. Infants were considered LR if they were nontoxic appearing, previously healthy, term infants with no focal source of infection upon examination. While the Baraff guidelines considered all infants less than 28d to be HR, we opted to only consider patients for LR stratification if they were older than 30 days. The laboratory criteria for LR patients included a WBC count of 5–15,000/mm^3^, <1500 bands/mm^3^, negative gram stain of unspun urine or <10 WBCs/hpf, and <5 WBCs/hpf in stool when diarrhea is present.

### Statistical Analysis

Statistical comparisons were performed using a two-tailed t-test and chi-square testing for all numerical and categorical values, respectively. Significance was set at p<0.05. We also performed a Bayesian analysis to calculate unconditional probabilities of SBI using a beta binomial with a (1,1) prior. This type of analysis generates an unconditional probability rather than a conditional one based on the null hypothesis being accepted or rejected [18]. Bayesian analysis also allows the incorporation of past studies/experience into current data analysis to generate a probability distribution that may be more clinically relevant. We generated probability curves for SBIs in the early and later periods and compared them to 2 other published SBI rates, the original Rochester study and a more recent study from Utah [5], [15]. We used SPSS Version 15.0, an open-source, web based program (www.openepi.com) and JMP7 (SAS) to perform the various statistical testing.

## Results

Between 1997–2006 1501 infants < = 90 days old had a blood culture drawn for any reason in our ED. The numbers of patients included in the final analysis are summarized in Table 1. Our screening process for FWLS looked first for significant PMH, then absence of fever, ill appearance, and focality of exam. We did include infants born as young as 36 weeks if there was documentation that they were routinely discharged and had no underlying medical problems. After review, 668 patients (44.5%) fit the criteria for FWLS and were included in the analysis. We divided the time period into two 5-year blocks (1997–2001 and 2002–2006) that roughly approximated the time before and after the transition from risk-based to culture-based screening for GBS by the Duke Department of Obstetrics and Gynecology. The earlier period included more infants in the youngest age group (≤30d), while the later period included more infants in the older age group (61–90 days). Otherwise the groups were not significantly different (Table 1). A total of 833 infants did not meet criteria for FWLS, 386 with significant PMH, 355 who were afebrile, 37 who were ill appearing, 29 with a focal exam, and 26 that had incomplete medical records. The majority of those with significant PMH were former premature infants with a previous NICU stay or infants born with a congenital anomaly (e.g. congenital heart disease, shunted hydrocephalus) or immunodeficiency (e.g. SCID, HIV exposure). Most of the remainder had either been hospitalized previously or were currently on antibiotics.

**Table 1:** Patient Demographics.

	1997–2001	2002–2006	*p* values
**Eligible Infants**	**735**	**766**	
**Included FWLS Infants**	**307**	**361**	
**Male/Female**	**169/138**	**211/150**	0.61
**0–30d**	**104**	**97**	0.05
**31–60d**	**134**	**151**	0.64
**61–90d**	**69**	**113**	0.01
**HR/LR**	**178/129**	**204/157**	0.70
**Blood Cx performed** *	**307**	**361**	
**UCx performed**	**259**	**321**	0.08
**LP Performed**	**233**	**235**	0.002

*initial inclusion criteria so by definition encompasses all subjects.

### Number and Rate of SBIs

**Figure 1:**
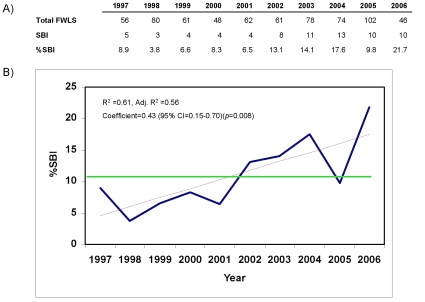
Analysis of Raw Data and Trends in SBIs among FWLS Infants, 1997–2006. A) Raw numbers of FWLS infants as well as SBIs and % SBI. B) Data in Figure 1a is graphed by %SBI on the y axis and year on the x axis (blue line). Also presented: overall mean %SBI (green line) and linear regression (red line). Statistical data on linear regression line is included.

The year by year results of our analysis are represented in Figure 1A. The overall rate of SBIs in our study over 10 years was 10.8%, but closer analysis of the trend line reveals a marked increase in SBIs during this ten year period (Figure 1B). When examined as a continuous variable, the rate of SBI in those infants ≤90d with a blood culture drawn increased over time (coef 0.43, p<0.008) and the goodness of fit of our trend line showed a modest association (R^2^ = 0.61). When examined as two distinct groups, infants in the later period had significantly more SBIs than those in the earlier period (14.4% vs. 6.5%, p = 0.001), with increased UTIs accounting almost entirely for the difference (45 cases of UTI in the later period vs. 13 in the earlier period, p = 0.002) (Table 2).

**Table 2:** Types of Serious Bacterial Infections in Early and Later periods.

	1997–2001	2002–2006	*p* value
**SBI**	20	52	0.001
**%SBI**	6.5%	14.4%	
**UTI**	13	45	0.0002
**Bacteremia**	7^a^	11^b^	0.54
**Meningitis**	1	2^c^	0.64

a one patient had UTI+Bacteremia.

b five patients had UTI+Bacteremia.

c one patient had Bacteremia+Meningitis.

**Figure 2:**
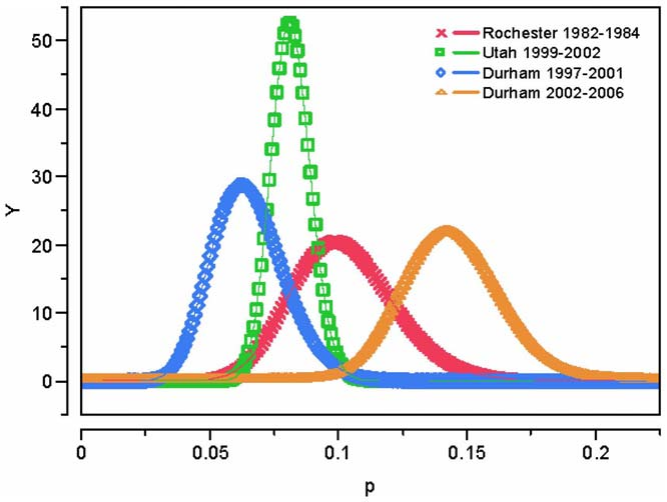
Bayesian Analysis of SBIs among FWLS Infants, 1997–2001 and 2002–2006. The graph represents the probability of SBI based on data from the early and later periods of this study compared with the Rochester data as well as data on FWLS infants from Utah [5], [15]. The probability curve from the data from the later period centers at approximately 14.4% whereas the other three curves all overlap between 5–10% probabilities.

Results from the Bayesian analysis comparing our data with that from the original Rochester study and a more recent study from Utah [5], [15] are presented in Figure 2. The probability curves for the Rochester and Utah studies overlap with the SBI probability from the early period of our study. The SBI probability curve for the later period of our study has very little overlap with the tail of one of the other curves and no overlap with the others, reflecting a distinct epidemiology among the infants in this group.

### Breakdown of SBIs by type and age group

During the period of 1997–2001, 4.2% of the sample had positive urine cultures, the majority of which were *E. coli* (84.6%) (Table 3). From 2002–2006, 12.2% of the sample had positive urine cultures (p = 0.0002), almost all of which were *E. coli* (91.5%). We did see patients that had two or more species of bacteria isolated from their urine. Unless two distinct pathogens are isolated, our lab reports multiple speciation as “mixed flora” and these are considered contaminants and were not included as positive UTIs. We did, however, count as positive two patients with urine cultures that met our inclusion criteria who had two pathogenic species isolated by urine culture (*Enterobacter* and *Proteus*; *E. coli* and *Proteus*). In the early period, one of the patients with UTI had a concordant bacteremia, compared with five patients in the later period, all of which were concordant *E. coli* from each site.

**Table 3:** Bacterial Pathogens by Species and Site of Infection.

	1997–2001				2002–2006			
Pathogen	Total # Pts	Blood	Urine	CSF	Total # Pts	Blood	Urine	CSF
**Gram-negative**								
*Escherichia coli*	11^a^	1	11	-	44	5^b^	43	1
*Enterobacter cloacae*	-	-	-	-	3	2	1^c^	1^d^
*Citrobacter* sp.	1	-	1	-	-	-	-	-
*Proteus mirablis*	-	-	-	-	3	-	3^e^	-
*Moraxella cattarhalis*	-	-	-	-	1	1	-	-
*Pasteurella multocida*	-	-	-	-	1	1	-	-
**Gram-positive**								
*Streptococcus pneumoniae*	1	1	-	-	-	-	-	-
*Staphylococcus aureus*	1	1	-	-	-	-	-	-
*Enterococcus* sp.	-	-	-	-	1	1	-	-
*Streptococcus agalactiae (GBS)*	5	4	1	-	1	1	-	-
*Listeria moncytogenes*	1	-	-	1	-	-	-	-

a One infant with *E coli* bacteremia had a concordant *E coli* UTI.

b 5 infants with *E coli* bactermias had concordant *E coli* UTIs.

c One infant with *Enterobacter* UTI had a concomitant *Proteus* UTI.

d One infant with *Enterobacter* meningitis also had a concordant *Enterobacter* bacteremia.

e One infant with *E coli* UTI had a concomitant *Proteus* UTI.

In 2002–2006, Total Patients with SBI = 54; Actual number is 52 – this discrepancy is due to the fact that one patient had a Enterobacter and Proteus UTI and one patient had a Proteus and E coli UTI.

There were 19 patients with a positive urine culture but <10 WBCs on the UA, 5 in the early period and 14 in the late period. In the early period, one of these patients met criteria for colonization (clean catch specimen with >100,000 *E. coli* but negative UA), and five patients from the later period met criteria for colonization (3 with *Enterococcus*, one *Pseudomonas*, and one *E. coli*).

There was no significant difference in episodes of bacteremia with 7 cases (2.3% or 23 cases per 1000 infants with FWLS) in the early period and 11 cases (3%) in the later period (p = 0.54) (Table 3). *E. coli* was responsible for 14.3% of cases in the early period and 45.5% in the late. During the period of 2002–2006, we saw 2 (0.6%) cases of meningitis. One of these was due to *E. coli*. The blood culture for this patient was negative, but multiple attempts to obtain a urine specimen were unsuccessful. The other case was caused by *Enterobacter cloacae* in a patient that also had a positive blood culture.

UTI rates were increased in all age groups, but the increase was not statistically significant in the youngest infants (≤30d) (Table 4). Between 1997–2001 and 2002–2006 the rate of UTI amongst infants ≤30d went from 3.8% to 7.2% (p = 0.3). Amongst those 31–60d it went from 3.7% to 11.9% (p = 0.01). In the infants age 61–90d the incidence of UTI increased from 5.8% to 17.7% (p = 0.02). There were no other significant differences between the early and late period by age group though there was a trend towards increasing bacteremia in the infants aged 0–60d.

**Table 4:** SBIs by Age Group.

Age	1997–2001				2002–2006				
	SBI	UTI	Bacteremia	Meningitis	SBI	UTI	Bacteremia	Meningitis	*p* value-UTI
**0–30 days**	7^a^	4	3	1	9^b^	7	5	-	0.30
**31–60 days**	8	5	3	-	22^c^	18	6	1	0.01
**61–90 days**	5	4	1	-	21	20	-	1	0.02

a 1 UTI was urosepsis.

b 3 UTIs were urosepsis.

c 2 UTIs were urosepsis, 1 meningitis had concomitant bacteremia.

### Ampicillin Resistance

Ampicillin resistance for all SBI was 41.7% over the 10 year period, 25% for the period 1997–2001 and 48.1% during the later period 2002–2006 (p = 0.08). Examining the UTI subset, the rate was 46.6% overall and, again, the difference between the early and late periods did not achieve statistical significance. Initial antibiotic choice was changed in a third of cases of SBI after antibiotic sensitivities were known. The six patients with ampicillin resistant gram negative rod bacteremia (1 from the early period, 5 from the later period) all had antibiotics switched when sensitivities were known and all had longer antibiotic courses compared to non-bacteremic infants. Four of those patients stayed at least 2 extra days in the hospital and the other two kept indwelling venous catheters for at least 2 extra days.

### Management of FWLS Infants

Management differed both over time and between age groups (Table 5). Among the age groups over the 10 year period almost all infants 30d or less received a complete workup including collection of blood, urine, and CSF cultures. There was no statistical difference in urine studies performed between the younger (≤30d) and older (31–90d) infants (p = 0.54), but older infants (31–90d) had significantly fewer LPs performed and antibiotics given than younger infants (p<0.001). Among those older infants that were stratified as HR, only 66.9% had an LP performed which is highly significant compared with the infants less than 30d old (p<0.001). Comparing 1997–2001 with 2002–2006, there were significantly fewer LPs performed (p = 0.002) and antibiotics given (p = 0.0004) in the later period.

**Table 5:** Management of Infants with FWLS in Early and Later periods.

1997–2006				
	0–30	31–60	61–90	All ages
**# patients**	201	285	182	668
**LP Performed**	188 (93.5)	202 (70.9)	78 (42.9)	468
**UCx Performed**	177 (88.1)	253 (88.8)	150 (82.4)	606
**Antibiotics given**	196 (97.5)	245 (86)	121 (66.5)	562
1997–2001				
# patients	104	134	69	307
LP Performed	97 (93.3)	104 (77.6)	32 (46.4)	233
UCx Performed	88 (84.6)	121 (90.3)	50 (72.5)	259
Antibiotics given	101* (97.1)	123** (91.8)	51** (73.9)	275
2002–2006				
# patients	97	151	113	361
LP Performed	91 (93.8)	98 (64.9)	46 (40.7)	235
UCx Performed	89 (91.8)	132 (87.4)	100 (88.5)	321
Antibiotics given	95 (97.9)	122# (80.8)	70∧ (61.9)	287

Comparing older infants (31–90d) vs. younger (≤30d):

fewer LP's performed (p<0.001);

no difference in UCx's performed (p = 0.54);

significantly fewer antibiotics given (p<0.001).

Comparing 2002–2006 vs. 1997–2001 overall (i.e. not broken down by age group):

significantly fewer LPs performed (p = 0.002);

no difference in UCxs performed (p = 0.08);

significantly less antibiotics given (p = 0.0004).

*Unknown if abx given to 1pt.

**Unknown if abx given to 2pts.

# Unknown if abx given to 1pt.

∧ Unknown if abx given to 3pts.

## Discussion

The purpose of this study was to investigate the rates of SBI, patterns of antibiotic resistance, and practitioner management, given the possible changing epidemiology. In our analysis, we identified several trends that may impact the care and outcomes of this group. The most significant was a major increase in the rate of SBI, arising predominantly from increased rates of *E. coli* UTIs which has not been previously reported. This increase is particularly pronounced when compared to previous studies. The original Rochester study had an SBI rate of 10%, but they included infants with a focal exam in their study which would have resulted in higher rates of SBI [5]. A more recent study from Utah demonstrated an 8% SBI rate [15]. Our 2002–2006 SBI rate of 14.4% is markedly higher. The reasons for this increase are not entirely clear, but we hypothesize several potential factors that may have contributed in some way to this increase.

The first is widespread intrapartum antibiotic prophylaxis (IAP) for GBS may be selecting for gram negative bacteria that have a propensity to cause disease in young infants. Many studies have examined IAP ampicillin use and found no increase neonatal *E. coli* sepsis [19], [20], [21], [22]. In contrast, two studies found that infants with invasive *E. coli* were more likely to have been exposed to IAP [23], [24]. Our hospital pharmacy data indicates that from 2002–2006, which are the earliest years available, approximately 20–30% of pregnant women received ampicillin. One would expect that if IAP was the primary reason for the increase, most of the cases would be among the youngest infants closest to the exposure during delivery. Our data shows the opposite, that the burden of UTI is spread disproportionately among the older infants.

Another potential reason is the emergence of a new clone that coincided with the switch in GBS prophylaxis at our institution. Clonal group A is known as a multidrug resistant uropathogenic group known to cause UTIs in adults [25]. Its epidemiology in infants is unknown. The other possibility is *E. coli* expressing the Dr adhesins that are usually associated with pregnancy related UTIs may have crossed into the infant population [26]. We are currently initiating studies of stored *E. coli* isolates to investigate this clonal possibility.

Another potential contributor is the lower rate of circumcisions over the past several years, which would increase a patient pool at higher risk of UTI [27], [28], [29]. NC Medicaid stopped paying for circumcision permanently starting November, 2002 with a resulting drop in the circumcision rate from 74% in 2000 to 40% in 2004 [30]. While this remains a possible explanation, it is noteworthy that male to female distribution of SBIs did not change significantly over the 10-year period.

Another possible explanation is sampling error and/or selection bias. We selected our patient population from those infants seen by physicians in the ED who had a blood culture drawn for any reason. It is possible that there were infants who presented to our ED with FWLS who did not have a blood culture drawn and if these infants did not subsequently develop an SBI that would mean our rates are artificially high. We also noted that there were more infants excluded in the early period compared to the later period for insufficient data. This is likely a reflection of the transitional years from a combined paper and electronic record to the current electronic one, but that difference could not explain the differences between the two periods.

Regardless of the reason, our results emphasize the need to sample urine on every infant less than 90 days of age. While practitioners are doing a better job at collecting urine studies, even at 93.4% of patients with a urine evaluation, with a 12.2% UTI rate in the 2002–2006 period out of the 361 FWLS infants, 2–3 UTIs were potentially missed.

The second important issue our results raise relates to antibiotic resistance. Ampicillin, in combination with gentamicin, has been traditionally used in the younger infants for its efficacy against *Listeria*, *Enterococcus*, and GBS. In 1999, Sadow *et al* found that the majority of pathogens were gram negative bacteria with high rates of ampicillin resistance [16]. They observed no cases of *Listeria* over a 4 year period. In 2003, Byington *et al* found similar results over a 3-year period [15]. In our study, 80% of disease was caused by *E. coli* with high rates of ampicillin resistance. We would argue that empiric antibiotics should cover the most likely invasive pathogen and appropriately account for the risk of antibiotic resistance. The vast majority of isolates causing UTI would no doubt respond to a regimen of ampicillin and gentamicin given the latter's utility in treating UTI. However, 8–9% of the SBIs in these infants would be a case of bacteremia or meningitis with an ampicillin resistant *E. coli* (5/52 from 2002–2006, 6/70 overall), a situation where most practitioners would not consider what effectively would be gentamicin monotherapy as appropriate. As a comparison, vancomycin plus cefotaxime/ceftriaxone is recommended in *S. pneumoniae* meningitis where the risk of cefotaxime/ceftriaxone non-susceptibility is approximately 7% [31]. We would suggest that careful attention be paid to local resistance rates in this infant population, and perhaps in a setting with resistance rates as high as ours, an empiric regimen of ampicillin and cefotaxime would appropriately cover virtually all likely pathogens in this patient population.

Our study also shows that practitioners perform much less aggressive evaluations on older infants. At first glance this may seem appropriate since these patients are old enough to be stratified as HR or LR and, if LR, receive a more limited evaluation. However, when the older infants were analyzed by risk according to the Baraff guidelines [17], we found that only 66.9% of HR patients received an LP, suggesting that guidelines are only inconsistently followed. Given the concern that UTI in this age group puts infants at risk for meningitis and our own observation that one of our infants was diagnosed with concomitant UTI and meningitis, it still seems reasonable that all high-risk patients should receive an LP.

Our findings should be analyzed in light of certain limitations. This is a retrospective study from a single center, and the volume and patient population seen in our ED may not be generalizable to larger practice settings. This study should be repeated at other institutions to see if the increase in UTIs is present. Our inability to determine the number of febrile children that did not receive a blood culture in the ED means that our frequencies of FWLS may be falsely high and our total rates of SBI also may be falsely high. This, in turn, limits our ability to draw comparisons with other studies, such as the Utah study, where data was collected prospectively on all infants with fever [15]. There are limitations with applying criteria retrospectively since some classification data may not have been recorded in the chart.

In this study, we have observed a significant increase in the number of SBI in infants with FWLS. These were primarily UTIs and the reasons for this are not yet clear. These results reinforce the need to examine the urine in all of these infants regardless of risk stratification and age. Our results also further suggest a move away from the combination of ampicillin and gentamicin as primary empiric therapy. The data from this study also may assist to streamline the management of these infants by helping to delineate risk differences in older versus younger infants.
